# The association between financial support of adult children to their parents and informal care provision in China and its differences in household registration, residence arrangement and community-based care services: 2008 ~ 2018

**DOI:** 10.1186/s12939-023-01856-z

**Published:** 2023-03-14

**Authors:** Hang Liang, Boyu Wang, Yanli Wu, Qilin Zhang, Nan Xiang, Zhang Yue, Erpeng Liu

**Affiliations:** 1 School of Public Administration, Zhongnan University of Economics and Law, Wuhan, 430073 China; 2 Center for Social Security, Wuhan University, Wuhan, 430072 China; 3 Institute of Income Distribution and Public Finance, Zhongnan University of Economics and Law, No. 182 Nanhu Rd, Wuhan, 430073 China

**Keywords:** Financial support, Informal care provision, Chinese elderly people, Longitudinal study

## Abstract

**Background:**

The changes in demographic and family structures have weakened the traditional norms of filial piety and intergenerational relationships dramatically. This study aims to examine the dynamic association between financial support of adult children to their parents and informal care provision in China and its differences in household registration, residence arrangement and community-based care services.

**Methods:**

Data was derived from the 2008–2018 Chinese Longitudinal Healthy Longevity Survey (CLHLS), which is a longitudinal survey of a nationally representative sample of individuals aged 60 and over. Random effects model was used to assess the association between financial support and informal care provision of adult children to their parents.

**Results:**

It was found that financial support showed an upward trend while informal care provision showed a download trend from 2008 to 2018. The result indicated a significant and negative association between financial support and informal care provision of adult children to their parents (B = -0.500, 95% confidence interval (CI) = -0.761 to -0.239). And the association was significant among elderly people who were from urban areas (B = -0.628, 95% CI = -0.970 to -0.287), co-resided with adult children (B = -0.596, 95% CI = -0.939 to -0.253), and had community-based services (B = -0.659, 95% CI = -1.004 to -0.315).

**Conclusion:**

Financial support was negatively associated with informal care provision of adult children to their parents in China, and the association has differences in household registration, residence arrangement and community-based care services. It is suggested that policymakers should prioritize planning interventions for elderly care services and establish a family caregiver support system.

## Background

China is undergoing a rapid population aging process. By the end of 2021, the number of Chinese elderly people over 60 had reached 267 million, accounting for 18.9% of the total population [1]. In addition, compared with the number of elderly people in 2000 when China became an aging society, the net increase of elderly population is approximately 138 million. It is estimated that by 2050, the number of elderly people over the age of 60 in China will rise to a peak of 488 million, representing 35.6% of the total population [2]. However, China is an unhealthy aging society. More than 30% of elderly people suffer from at least one kind of chronic disease, and more than 15% of elderly people need care services provided by others [3, 4]. At the same time, China established a primary pension insurance system to cover elderly people, but the pension is low and is growing slowly. As many as 150 million Chinese elderly people receive a monthly pension from 100 to 200 CNY (about 16 ~ 32 $) [5], which means that if there is no financial support from adult children, the income of most elderly people will not exceed the poverty line of 2,300 CNY set by the state in 2016. A study showed that the poverty rate among elderly people in China reached 30% [6], which highlights the importance of adult children’s financial support in reducing the risk of poverty among elderly people.

In the transformation from a traditional society to a modern one, China is experiencing a decline in fertility rate, a shrinking in family size, a separation in intergenerational residence arrangement, and the reversal of intergenerational family status. According to statistics, the fertility rate in China dropped from 5.81 in the 1970s to approximately 1.15 in 2021 [7]. The average family size is only 2.62, and the proportion of one-person household and two-person households exceed 50% [8]. Empty nesters accounted for more than 50% of elderly people’s households, and 13.1% of elderly people lived alone in 2015 [3]. During this period, the way in which elderly people receive care services has gradually changed from single informal care provided by family members such as adult children and spouses to a “mixed pattern” that includes informal care provision and a small amount of formal care provision, but informal care is still the mainstay of elderly care services [9, 10]. In China, the type of formal care mainly includes paid care services provided by nursing homes, hospitals and home care staffs. These formal care provisions are open to all elderly people. Since 2015, after the government provided subsidies of long-term care, the general price structure of formal care was revised to 170 CNY to 200 CNY per day for care in hospitals, 65 CNY per day for nursing homes, 50 CNY per day for home care staffs [11]. Elderly people with better economic conditions, especially those in urban areas, could purchase market-based care services to meet their needs for personalized care services [12]. China has launched a series of policy initiatives to develop a system of services for elderly people, which consists of three tiers of social services: home-based care as the “basis”, community-based services as “backing”, and institutional care as “support” [13]. In particular, formal care has developed dramatically over the years. On the one hand, the Chinese government has begun to strengthen the construction of facilities for community-based care services since 2000 [14]. By the end of 2021, the facilities of community-based care services reached 318,000, and community-based care services are usually funded by local government and free to community residents [15]. On the other hand, China’s market-based elderly care services are also gradually developing. By 2016, the GDP of the market-based elderly care and nursing services reached 57 billion CNY [16]. There has been approximately 70% growth in the number of care home beds (6.7 million), reaching the target of 30 beds per 1000 elderly people [17].

Studies in some developed countries have demonstrated that there are substitutions between informal and formal care. In Germany, long-term care policies provide cash benefits, which made family caregivers reduce the time of home care, increase labor supply, and pay more formal care for older people [18]. Long-term healthcare services have reduced informal care from family members in the Nordic countries, such as Norway and Sweden, but population ageing and strained public resources will likely challenge the future provision of formal old-age care [19]. In contrast, other studies estimate a complementary effect, which found that formal care increases the provision of informal care [20]. A similar complementary effect of community and informal care is also observed in Japan [21]. In China, there seems to be a substitution between formal and informal care, and the use of formal care services for elderly people is regarded as a sign of the decline of the traditional filial piety culture and the informal care model by some studies [22, 23]. However, more studies have shown that: First, adult children (including daughters-in-law and sons-in-law) are still the main providers of care services for their parents, and that more than 80% of care services come from elderly people’s adult children, especially in rural areas where formal care services are underdeveloped [24]. Second, adult children’s financial support constitutes the most important source of income for the majority of their parents and has become an important way to compensate for low pensions income [25]. In addition, although there is a trend of separation of intergenerational co-residence arrangement, most of elderly people and their adult children still live closely to each other in same communities or villages [26, 27]. Therefore, financial support and informal care provision of adult children to their parents will not disappear, but instead, they will remain as a strong connection which is compatible with modernization.

Financial support and informal care provision are two core contents of adult children’s support to their parents, and they are also indispensable resources to ensure the happiness of elderly individuals. In the decision making within the family, the association between financial support and informal care provision is changing and its ultimate goal is to maximize the benefits of the family [28]. However, there are some obvious differences between financial support and informal care provision. Taking care of parents, especially who come with poor physical conditions, requires more time, energies and patience from their adult children, which made negative influence on the physical condition, income and leisure of adult children [29, 30]. Compared with informal care provision, financial support is easier to perform under the background of the shrink in family size, frequent population mobility, increasingly fierce labor market competition and improved accessibility of formal care services [31, 32]. Some scholars pointed out that intergenerational relationships have shifted from reflecting filial piety in terms of both financial support and care-giving from younger generations to increasing financial transfers accompanied by a decreasing responsibility of informal care provision [33, 34]. And recent research found that financial support from the younger generation was negatively associated with the informal care provision [35]. Based on that, we propose the following questions: What is the association between financial support and informal care provision of adult children to their parents in China? What is the trend of this association? What are the group differences in this association?

Although previous studies found that there is a negative association between financial support and informal care provision, they are mostly based on cross-sectional data or data in some regions, and some studies mainly focus on a particular group, such as elderly people with a disability or dementia or living in cities [35, 36]. It is necessary to investigate the relationship between financial support and informal care provision from a dynamic perspective using multi-period data, which will make the research conclusions more reliable. In addition, there is strong heterogeneity within Chinese elderly people, such as groups of different household registration, residence arrangement and the availability of community-based care services. Household registration in China determines eligibility for various welfare benefits, such as education, health insurance, pension insurance, housing and employment, which has led to a huge difference in social welfare and economic status between urban and rural residents [37]. Co-residence represents an important source of informal care, and elderly people living alone are much more likely to rely on formal care, while those who live with adult children receive more informal care [19]. Currently, China has vigorously developed community-based care services such as housekeeping services, rehabilitation care and spiritual comfort services in order to make up for the lack of informal care. However, the services provided at community centers varied greatly in quality and quantity, some communities even do not provide services for elderly people [38]. There were group differences in the association between financial support and informal care provision of adult children to their parents, which has not been considered in previous studies and deserve further study. The marginal contributions of this study are as followed: First, we described the trends of financial support and informal care provision of adult children to their parents from 2008 to 2018. Second, the dynamic association between financial support and informal care provision of adult children to their parents was explored by using longitudinal data from 2008 to 2018, which overcomes the limitations of research based on cross-sectional data. Third, we analyzed the difference of household registration, residence arrangement and the availability of community-based care services in the association between financial support and informal care provision form adult children to their parents.

## Methods

### Data sources

The data was derived from the Chinese Longitudinal Healthy Longevity Survey (CLHLS), which was a nationally representative survey performed jointly by the Center for Healthy Aging and Development Studies at Peking University and Duke University. A baseline survey was conducted in 1998 and was followed by seven waves of surveys in 2000, 2002, 2005, 2008, 2011, 2014 and 2018, which was from 22 sample areas in 31 provincial administrative units. The population of the surveyed region accounts for 85.3% of the total population in China, which could be regarded as a nationally representative sample [39]. All respondents were tracked in the later waves unless death or loss to follow-up occurred, and the response rates are 88 to 90% in the waves of CLHLS [40]. The Ethics Committee of Peking University approved the CLHLS study (IRB00001052-13,074). Additional details, such as the sampling design, sampling weight and assessment of data quality, can be found in previous studies [41, 42].

**Fig. 1:**
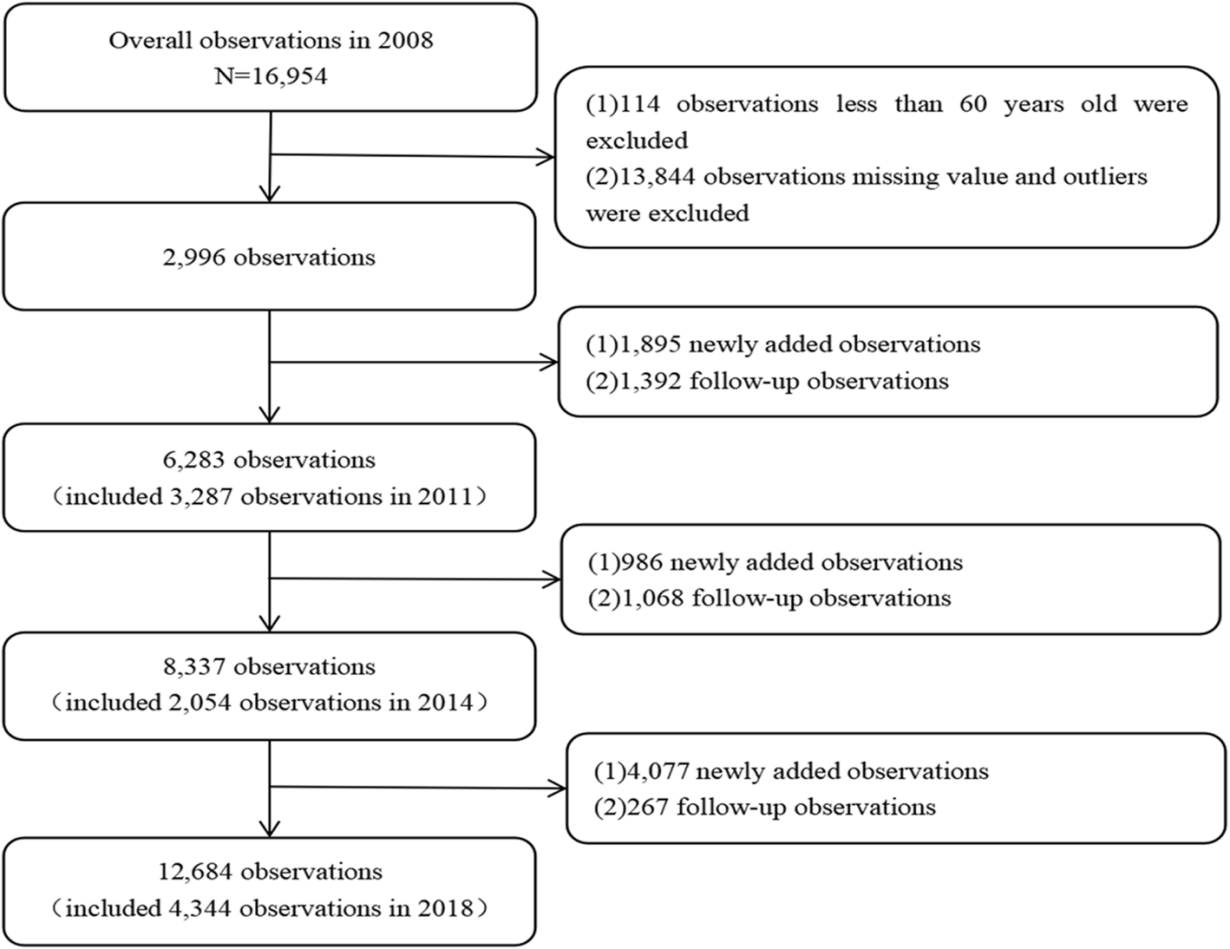
Flowchart describing the sample selection

This study employed the longitudinal sample from 2008 to 2018, which contained rich information on financial support, informal care provision, health status and lifestyle, family resources, adult children’s information, community-based care services, as well as characteristics of the respondents. After excluding cases with missing information on key variables and considering that there is insufficient information to confirm the age reported by extremely elderly people, we also excluded elderly people aged 120 and over. The final sample (See Fig. 1) consisted of 2,996 elderly people aged 60 and over in the 2008 wave, 3,287 in the 2011 wave, 2,054 in the 2014 wave, and 4,344 in the 2018 wave. And there were 9,402 respondents who participated in one wave, accounting for 74.14%, 2,665 respondents who participated in two waves, accounting for 21.02%, 597 respondents who participated in three waves, accounting for 4.71%, and 17 respondents who participated in four waves, accounting for 0.13%. The study sample selection process is shown in Fig. 1.

### Measures

#### Dependent variable: informal care provision

The dependent variable is informal care provision. In the CLHLS, the respondents were asked “How many hours did your children and their spouses spend in providing care to you last week in total?”. That reflects the received time of informal care of the elderly. The informal care provision variable ranged from 0.5 to 168 h. We subsequently categorized informal care provision into three groups: high, medium and low intensities. High intensity of informal care provision is defined as receiving more than 50 h of informal care per week, medium intensity is defined as receiving between 21 and 50 h of informal care per week, while low intensity is defined as receiving 20 h or less of informal care per week. The classification method of informal care intensity is based on a number of previous studies [43, 44].

#### Independent variable: financial support

The independent variable is financial support. Financial support is measured by the amount of cash and value of materials received from adult children. In the CLHLS, participants were asked the following questions: “How much money (including cash and material objects) did you get from your children and their spouses no matter living with you or not last year?”. After eliminating the extreme values, we subjected logarithmic processing of financial support. We had adjusted financial support for inflation during processing the data.

#### Covariates

All analyses included a series of covariates associated with the care recipient according to the previous studies [19, 25, 36, 45, 46]. The sociodemographic characteristics included gender, age, marital status, education level, household registration. Health status and lifestyle included chronic disease, activities of daily living (ADL), smoking, alcohol consumption and exercise. ADL included the six items of eating, bathing, dressing, bathroom use, getting in and out of bed, and moving around indoors. For each item, the respondents were provided with three choices (not difficult at all = 1, slightly difficult = 2, and unable to perform the task = 3). The total ADL scores ranged from 6 to 18, and the higher scores indicate more limited function of the respondent. Family resources included the number of adult children and family income (natural logarithm). Bargaining power included whether the respondents owned real estate in their own name, whether the income is sufficient for paying daily expenses, and the frequency of performing housework. Intergenerational relationships included residence arrangement and frequency of communication with adult children. Residence arrangement were assessed by whether elderly people co-resided with adult children. Social services included medical insurance, pension insurance, and the types of community-based care services (0–8). There are eight types of community-based care services included personal daily care services, home visits, psychological consulting, daily shopping, social and recreation activities, legal aid, health education, and neighboring relations. These community-based care services are funded by local government and provided free for community residents.

### Statistical analysis

We first used descriptive analysis to describe the general characteristics of the participants and examined the trends in financial support and informal care provision of adult children to their parents from 2008 to 2018. Then, we assessed the association between financial support and informal care provision of adult children to their parents by random effects models. Finally, considering that there are differences in household registration, residence arrangement and the availability of community-based care services among elderly people, we explored the association between financial support and informal care provision in different groups. All analysis was conducted using the statistical software Stata15 (Stata Corp, College Station, TX, USA).

## Results

### Sample characteristics

Table 1 shows the characteristics of the participants from 2008 to 2018. The average financial support (natural logarithm) of adult children to their parents was 6.07. The average informal care provision of adult children to their parents was 46.39. The low intensity of informal care provision accounted for 48.4%, the medium intensity of informal care provision accounted for 22.33%, and the high intensity of informal provision accounted for 29.27%. Women accounted for 62.47% in the participants. The average age of the participants was 91.10. The average education years of the participants was 1.99. The participants who are single/divorced/widowed accounted for 75.56% in the participants. And individuals from rural areas accounted for 47.79% in the participants. Most of the participants come with the following features, which had chronic diseases, no ADL limitations, no alcohol consumption, two or more adult children, low family income, no real estate in own name, sufficient income for paying daily expenses, medical insurance and had no pension insurance. And most of the participants didn’t smoke, exercised, performed housework rarely or never, co-resided with adult children, often communicated with adult children and had fewer types of community-based care services.

**Table 1:** Descriptive statistics of the whole study sample (*N* = 12,681)

**Variables**	**Measurement**	**n (%)/mean (SD)**
Financial support (natural logarithm)	Continuous measurements	6.07 (2.23)
Informal care provision (hours)	Continuous measurements	46.39 (53.10)
	Low intensity (less than 20 h)	6138 (48.40%)
	Medium intensity (21 to 50 h)	2831 (22.33%)
	High intensity (more than 50 h)	3712 (29.27%)
**Sociodemographic characteristics**		
Gender	Female = 0	7,922 (62.47%)
	Male = 1	4,759 (37.53%)
Age	Continuous measurement	91.10 (10.08)
Education level (years)	Continuous measurement	1.99 (3.49)
Marital status	Single/divorced/widowed = 0	9,582 (75.56%)
	Married = 1	3,099 (24.44%)
Household registration	Rural = 0	6,060 (47.79%)
	City = 1	6,621 (52.21%)
**Health status and lifestyle**		
Chronic diseases	No = 0	5,158 (45.63%)
	Yes = 1	7,523 (59.32%)
ADL	Continuous measurement	8.95 (3.44)
Smoking	No = 0	11,192 (88.26%)
	Yes = 1	1,489 (11.74%)
Alcohol consumption	No = 0	11,168 (88.07%)
	Yes = 1	1,513 (11.93%)
Exercise	No = 0	9,909 (78.14%)
	Yes = 1	2,772 (21.86%)
**Family sources**		
Number of children	Have no children = 0	218 (1.72%)
	Have one child = 1	754 (5.95%)
	Have two or more children = 2	11,709 (92.33%)
Family income (natural logarithm)	Continuous measurement	9.75 (1.61)
**Bargaining power**		
Have real estate in own name	No = 0	8,199 (64.66%)
	Yes = 1	4,482 (35.34%)
Have sufficient income for paying daily expenses	No = 0	2,397 (18.90%)
	Yes = 1	10,284 (81.10%)
Frequency of performing housework	Rarely or never = 1	8,998 (90.96%)
	Sometimes = 2	377 (2.97%)
	Monthly = 3	154 (1.21%)
	Weekly = 4	578 (4.56%)
	Daily = 5	2,574 (20.30%)
**Intergenerational relationship**		
Residence arrangement	Lived alone = 0	4,054 (31.97%)
	Co-resided with adult children = 1	8,627 (68.03%)
Frequency of communication with children	Never = 0	3,399 (26.80%)
	Sometimes = 1	3,855 (30.40%)
	Often = 2	4,294 (33.86%)
	Always = 3	1,133 (8.93%)
**Social services**		
Medical insurance	No = 0	2,466 (19.45%)
	Yes = 1	10,215 (80.55%)
Pension insurance	No = 0	7,968 (62.83%)
	Yes = 1	4,713 (37.17%)
Community-based care services	Continuous measurement	1.39 (1.85)

Continuous variables are presented as means and standard deviations (SD)

*ADL* Activities of daily living

### The trends of financial support and informal care provision from 2008 to 2018

**Fig. 2:**
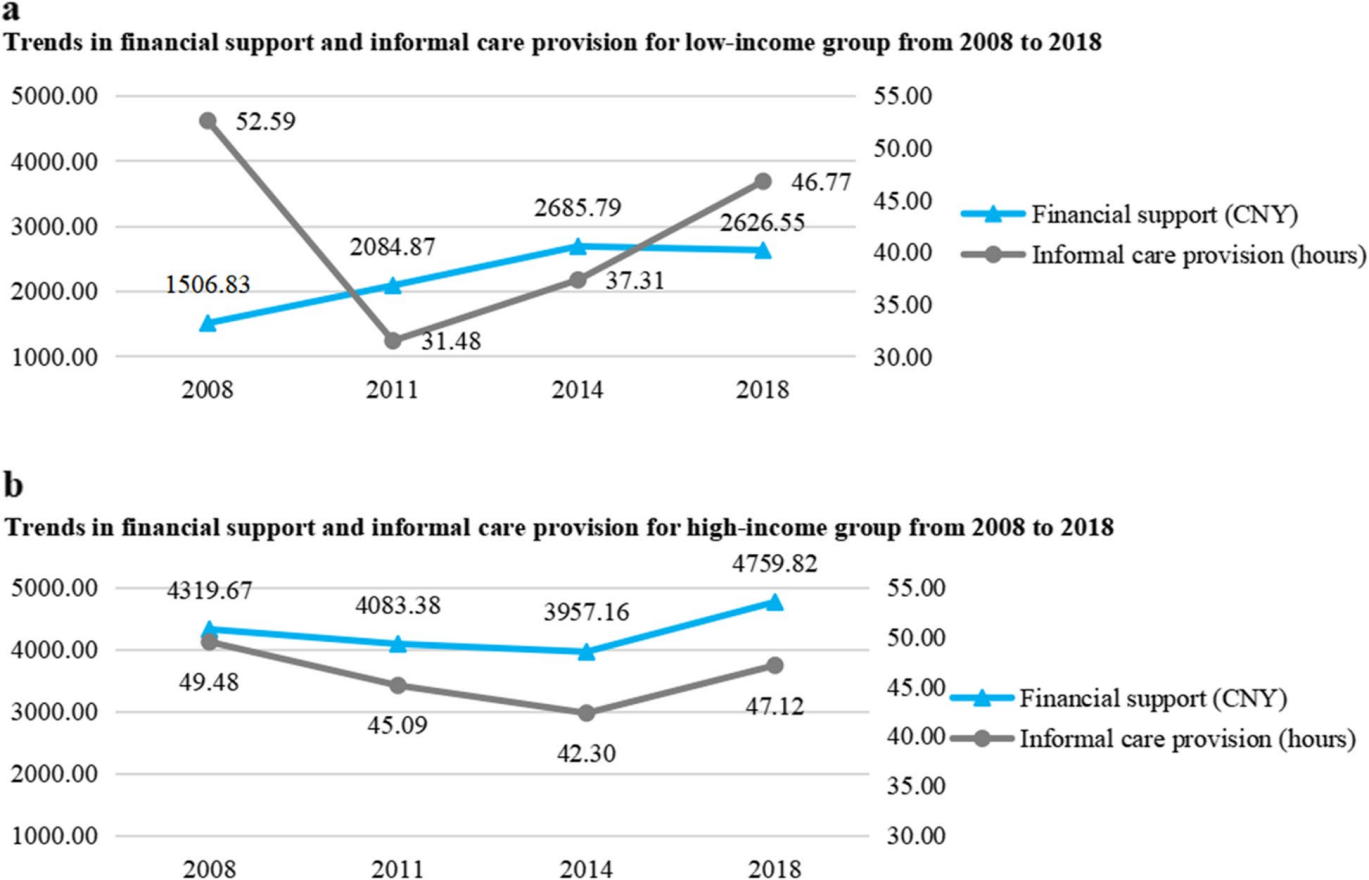
The trends of financial support and informal care provision for low-income and high-income groups from 2008 to 2018. Note: The left side of the figure shows units of financial support and the right-side shows units of informal care provision

There are differences in the family economic status of Chinese elderly people. We divided the family income of elderly people into ten equal parts, and considered the top 10 percent as the high-income group and the bottom 10 percent as the low-income group [47, 48]. Then, we investigated the trends of financial support and informal care provision of adult children to their parents among the two groups from 2008 to 2018. Figure 2a shows the trends in financial support and informal care provision from 2008 to 2018 among low-income group of elderly people. Although the financial support of adult children to their parents reduced slightly from 2014 to 2018, it showed an upward trend from 2008 to 2014 generally, and the average of financial support in 2018 increased by 1119.72 CNY compared with that in 2008. Informal care provision of adult children to their parents showed a downward trend from 2008 to 2018, and the average of informal care provision in 2008 decreased by 5.82 h compared with that in 2018. Figure 2b shows the trends in financial support and informal care provision from 2008 to 2018 among high-income group of elderly people. On the whole, financial support of adult children to their parents showed a rising trend from 2008 to 2018, and the average of financial support in 2018 increased by 440.15 CNY compared with that in 2008. Informal care provision of adult children to their parents showed a downward trend from 2008 to 2018, and the average of informal care provision in 2008 decreased by 2.36 h compared with that in 2018. It could be found that adult children provided less informal care provision and more financial support to their parents among low-income and high-income group of elderly people.

### The association between financial support and informal care provision

Table 2 shows the results of the random effects models, which indicated that there was a significant and negative correlation between financial support and informal care provision of adult children to their parents (B = -0.500, 95% confidence interval (CI) = -0.761 to -0.239) after adjusting for the covariates. We also found that some covariates were significantly related to informal care provision. Respondents who were female (B = -2.066, 95% CI = -0.015 to -4.116), were older (B = 0.310, 95% CI = 0.200 to 0.423), had chronic diseases (B = 3.347, 95% CI = 1.593 to 5.101), had worse ADL (B = 5.601, 95% CI = 5.315 to 5.886), had real estate in own name (B = 3.790, 95% CI = 1.789 to 5.790), had sufficient income for paying daily expenses (B = 2.744, 95% CI = 0.558 to 4.931), co-resided with children (B = 11.151, 95% CI = 9.060 to 13.248), and had pension insurance (B = 3.109, 95% CI = 1.183 to 5.036) were more likely to receive more informal care provision from their adult children. Compared with elderly people in rural areas, those in urban areas were more likely to get less informal care provision from their adult children (B = -3.546, 95% CI = -5.335 to -1.758). Elderly people who performed housework frequently (B = -1.606, 95% CI = -0.2.232 to -0.980) and had medical insurance (B = -2.349, 95% CI = -4.493 to -0.204) were more likely to get less informal care provision from their adult children. Time variable was significantly and positively with informal care provision (B = 1.252, 95% CI = 1.026 to 1.477).

**Table 2:** Longitudinal results of the association between financial support and informal care provision (*N* = 12,681)

**Independent variable**	**B**	**95% CI**	***p*-value**
Financial support	-0.500***	(-0.761, -0.239)	< 0.001
Gender	-2.066*	(-0.015, -4.116)	0.048
Age	0.310***	(0.200, 0.423)	< 0.001
Education level (years)	0.091	(-0.194, 0.377)	0.530
Marital status	-2.100 +	(-4.570, 0.370)	0.096
Household registration	-3.546***	(-5.335, -1.758)	< 0.001
Chronic diseases	3.347***	(1.593, 5.101)	< 0.001
ADL	5.601***	(5.315, 5.886)	< 0.001
Smoking	0.931	(-1.861, 3.722)	0.513
Alcohol consumption	-0.353	(-3.057, 2.350)	0.798
Exercise	-2.012 +	(-4.219, 0.195)	0.074
Number of children	0.102	(-1.300, 1.504)	0.887
Family income	-0.192	(-0.752, 0.368)	0.502
Have real estate in own name	3.790***	(1.789, 5.790)	< 0.001
Have sufficient income for paying daily expenses	2.744*	(0.558, 4.931)	0.014
Frequency of performing housework	-1.606***	(-2.232, -0.980)	< 0.001
Residence arrangement	11.154***	(9.060, 13.248)	< 0.001
Frequency of communication with children	0.112	(-0.793, 1.018)	0.808
Medical insurance	-2.349*	(-4.493, -0.204)	0.032
Pension insurance	3.109**	(1.183, 5.036)	0.002
Community-based care services	0.102	(-0.360, 0.564)	0.664
Time	1.252***	(1.026, 1.477)	< 0.001

*B* B-coefficient, *CI* Confidence interval, *ADL* Activities of daily living

^***^ *p* < 0.001, ** *p* < 0.01, * *p* < 0.05, and + *p* < 0.10

### The association between financial support and informal care provision among different groups

Table 3 shows the results of the difference in the association between financial support and informal care provision based on the household registration, residence arrangement and community-based care services. First, the association between financial support and informal care provision of adult children to their parents was significant in urban areas (B = -0.628, 95% CI = -0.970 to -0.287), but this association was not significant in rural areas. Second, the association between financial support and informal care provision of adult children to their parents was significant among elderly people who co-resided with adult children (B = -0.596, 95% CI = -0.939 to -0.253), but this association was not significant among those who lived alone. Third, the association between financial support and informal care provision of adult children to their parents was significant among elderly people who had community-based care services (B = -0.659, 95% CI = -1.004 to -0.315), but the association was not significant among those who had no community-based care services.

**Table 3:** The differences in the association between financial support and informal care provision based on the household registration, residence arrangement and community-based care services

	**Urban areas (*N* = 6621)**			**Rural areas (*N* = 6060)**		
	**B**	**95% CI**	***p*-value**	**Coefficient**	**95% CI**	***p*-value**
Financial support	-0.628***	(-0.970, -0.287)	< 0.001	-0.352 +	(-0.759, 0.056)	0.091
Covariates	Control			Control		
	**Co-resided with adult children** **(*N* = 8627)**			**Lived alone (*N* = 4054)**		
	**B**	**95% CI**	***p*-value**	**Coefficient**	**95% CI**	***p*-value**
Financial support	-0.596**	(-0.939, -0.253)	0.001	-0.362 +	(-0.726, -0.001)	0.051
Covariates	Control			Control		
	**Had community-based care services** **(*N* = 6711)**			**Had no community-based care services** **(*N* = 5970)**		
	**B**	**95% CI**	***p*-value**	**Coefficient**	**95% CI**	***p*-value**
Financial support	-0.659***	(-1.004, -0.315)	< 0.001	-0.293	(-0.690, 0.104)	0.148
Covariates	Control			Control		

*B* B-coefficient, *CI* Confidence interval

^***^ *p* < 0.001, ** *p* < 0.01, * *p* < 0.05, and + *p* < 0.10

## Discussion

Based on the national representative sample with longitudinal data, this study attempted to focus on all Chinese elderly people, exploring the association between financial support and informal care provision of adult children to their parents, and analyzing this association based on the differences in household registration, residence arrangement, and community-based care services. The results indicated that financial support showed an upward trend while informal care provision showed a download trend on the whole. It was found that financial support was negatively and significantly associated with informal care provision of adult children to their parents. And the association was significant among elderly people who were from urban areas, co-resided with adult children, and had community-based care services.

It was found that financial support was negatively associated with informal care provision of adult children to their parents, and financial support is increasing with the downward trend of informal care provision for low-income and high-income groups of elderly people. The results were consistent with previous studies [35], which indicated there is negative association between financial support and informal care provision. Economic reforms and labor-related migration have both improved the economic status of adult children, enhancing their capacity of offering more financial support to their parents, as the children’s disposable resources have increased [49]. The informal care provision in practice could be eroded due to the mobility and lack of availability of adult children [50]. The corporate group model has suggested that constrained by the decision to maximize family utility, there may be an alternative association between financial support and informal care provision of adult children to their parents [35, 51]. That is, adult children reduce the informal care provision by providing more financial support to their parents. On the one hand, the financial support from adult children promotes parents’ ability to purchase formal care services in the market and meet their care needs. On the other hand, adult children also have sufficient time to meet their career, income and leisure needs to maximize their own utility. As family structures change and formal care develops, more adult children will choose to provide financial support to their parents and reduce informal care provision [35].

It was found that there was difference of household registration in the association between financial support and informal care provision of adult children to their parents. For elderly people in urban areas, financial support was negatively and significantly associated with informal care provision of their adult children, while the association is not significant for those in rural areas. That could be explained by the differences in concepts of filial piety, pensions and the availability of formal care services caused by the dual structure of urban and rural areas in China. In rural areas, filial piety emphasizes the importance of informal care provision of adult children to their parents while filial piety is gradually declining in cities with a higher level of modernization [10]. In urban areas, the formal care service system (public care services and market-based care services) is gradually being established and improved, particularly elderly people in urban areas usually enjoy a relatively a high level of monthly pension which enable them to purchase more formal services [52, 53]. In contrast, in rural areas, due to the lower-level pensions and the lack of formal care service, the elderly people can only rely on the support from adult children to meet their own economic and care needs [24, 25].

The results showed that there was difference of residence arrangement in the association between financial support and informal care provision of adult children to their parents. The association of financial support and informal care provision was significantly negative among elderly people co-resided with adult children, but the association was not significant among those who lived alone. This suggests that adult children are more likely to replace informal care provision with financial support, even if they co-reside with their parents, which could be explained in two ways. On the one hand, elderly people who co-reside with adult children have more intergenerational communication, and get more filial piety from adult children [26]. It is obvious that financial support is an easier way to express filial piety, which could meet elderly people’s daily economic needs [12]. On the other hand, in China, many elderly people who co-reside with adult children tend to provide intergenerational care to their grandchildren, which makes adult children return the favor in the form of financial support.

There was difference of community-based care services in the association between financial support and informal care provision of adult children to their parents. For elderly people who had community-based care services, financial support was negatively and significantly associated with informal care provision while the association was not significant for those who had no community-based care services. The welfare triangle theory showed that the public care services provided by the government will have a crowding out effect on informal care provision [54, 55]. The provision of basic public care services in China is developing rapidly, and the supply of community-based care services brought Chinese family more alternative ways of elderly care services [48]. Many adult children could provide financial support rather than informal care provision to their parents when community provided some elderly care services [56]. As a result, community-based care services make crowding out effect of informal care provision of adult children to their parents, and the association between financial support and informal care provision of adult children to their parents was significantly negative among elderly people who had community-based care services.

Evaluating the association between financial support and informal care provision of adult children to their parents is beneficial for constructing a perfect care system aimed at providing and improving elderly care services (public care services and market-based care services) outside the family. Numerous elderly people in China, especially in rural areas, are confronting the dilemma of being left at home with nobody to take care of them. In consideration of the association between financial support and informal care provision varies in terms of the household registration, residence arrangement, and community-based care services, the Chinese government should pay more attention to the planning of the elderly care service system, expand the coverage of community-based care services and establish a support system for family caregivers. First, Chinese government needs to mobilize social resources to establish long-term care programs. Policymakers should leverage available policy instruments such as tax exemptions and subsidies, to explicitly encourage the construction of long-term care facilities, especially those in rural areas [57]. Second, supervise and manage the supply of community-based care services. To promote the development of community stations that provide daycare and temporary nursing services. Third, it is suggested that provide financial, emotional and physical health support for home caregivers. In particular, financial and care assistance should be provided for disabled elderly and lower-income households.

Nevertheless, the research findings from this article should be interpreted cautiously due to some limitations. First, the indicator of informal care provision was self-reported by the elderly people. The elderly people who co-resided with adult children may overstate the length of time about informal care provision, which would cause measurement inaccuracy of informal care provision. Second, some characteristics of adult children, such as occupation and working status, are important factors affecting the association between financial support and informal care provision. Unfortunately, these factors are not included in the CLHLS and we cannot select these variables in analysis. Last, due to the lack of detailed data on the financial support and informal care provision provided by each adult child, the distribution of financial support and informal care among each adult child was not fully considered in this study. Despite these limitations, this study discovered that the association between financial support and informal care provision of adult children to their parents was negative and significant, which provide evidence from Chinese families.

## Conclusion

This study demonstrated that financial support was negatively and significantly associated with informal care provision from the dynamic perspective, which fills the gap in literature on the association between financial support and informal care of adult children to their parents in China. It was found that the association of financial support of adult children to their parents and informal care provision was significant among elderly people who are from urban areas, co-resided with adult children and had community-based care services. But the association was not significant among elderly people who are from rural areas, lived alone and had no community-based care services. Considering the trends of decreasing informal care provision with increasing financial support of adult children to their parents, the social policies and social services should be initiated to support families. Policymakers should prioritize planning interventions for elderly care services and establish a family caregiver support system.

## Data Availability

CLHLS data are available at https://opendata.pku.edu.cn/dataverse/CHADS (requiring a simple application).

## Abbreviations

ADL: Activities of daily living
B: B-coefficient
CI: Confidence interval
CLHLS: Chinese Longitudinal Healthy Longevity Survey
